# Self-compassion, well-being, and body image concern in young Japanese women: The role of positive and negative facets of self-compassion

**DOI:** 10.1371/journal.pone.0334753

**Published:** 2025-11-12

**Authors:** Eriko Takahashi, Ryotaro Fukuda, Takumi Takahashi, Taisuke Katsuragawa

**Affiliations:** 1 Faculty of Human Sciences, Waseda University, Saitama, Japan; 2 School of Medicine, University of California, Davis, California, United States of America; 3 Student Support Center, Tokyo Denki University, Tokyo, Japan; National Institutes of Health, University of the Philippines Manila / De La Salle University, PHILIPPINES

## Abstract

The present study comprised two studies to examine how decreasing the negative facet of self-compassion, in addition to increasing its positive facet, is associated with body image and well-being in young Japanese women. Study 1 employed cross-sectional structural equation modeling using survey methodology to test hypothetical models. Participants (N = 577, mean age = 25.1 years, SD = 5.1) were recruited from an online community. A comparison of self-compassion models indicated that both one-factor and two-factor structures were valid, with the one-factor model and the negative component of the two-factor model significantly predicting well-being and appearance schemas, which in turn predicted body image concerns and abnormal eating behaviors. While these findings support the study’s hypothesized model and align with the cognitive-behavioral model of body image, the positive component showed only weak and maladaptive associations with body image variables, contrasting with prior intervention studies that reported beneficial effects. Study 2 was a parallel-group randomized controlled trial with 158 women randomly assigned to one of three groups: (1) decreased negative facet of self-compassion (DN; N = 48), (2) DN combined with increased positive facet (N = 53), or (3) non-intervention control (N = 57). After an image task to induce body dissatisfaction, participants completed measures of body dissatisfaction, self-esteem, and emotions before and after the self-compassion micro-intervention. The combined intervention significantly reduced body dissatisfaction and improved self-esteem compared to the control group. However, no significant difference was found between the DN-only and combined intervention groups. These findings suggest that the positive and negative facets of self-compassion have distinct effects on body image, well-being, and related outcomes. Further research is needed to clarify the mechanisms through which interventions that both reduce negative and enhance positive facets of self-compassion improve body image and emotional well-being.

## Introduction

In modern Japan, the body has become socialized and materialized, and Japanese individuals are constrained by appearances [1]. Body image has become a global mental health concern [2]. Concerns about body image are prevalent, particularly among younger women in Western societies and countries undergoing Westernization, such as Japan [3,4]. Body image concerns include excessive preoccupation with physical appearance, which often results in psychological distress and dysfunctional behaviors [5]. Such behaviors include unhealthy dieting practices [3,4,6,7], careful and repeated scrutiny of one’s body [8], social withdrawal [9], and other avoidance and safety behaviors related to physical appearance [5]. Based on the cognitive-behavioral theory of body image [10], appearance schemas generate and maintain body image concerns [11–14]. Appearance schemas refer to the extent to which an individual values physical attractiveness. Appearance schemas represent the psychological investments in appearance [10]. Notably, research on young Japanese women shows that appearance schemas have direct and indirect effects on self-esteem, happiness, and maladaptive behaviors, such as disordered eating, appearance-related avoidance (e.g., avoiding mirrors), and safety behaviors (e.g., excessive make-up) by mediating body dissatisfaction [15]. Thus, maintaining a good body image is important for psychological well-being.

Self-compassion is effective in increasing well-being [16], but its mechanisms are not fully understood. Self-compassion interventions aimed at reducing body image concerns and eating disorder symptoms have been shown to be effective [17,18]. Self-compassion significantly predicts body image concern and eating pathology, and shame is a full mediator of these relationships [19]. Self-compassion is defined as directing compassion inwardly [20], and comprises six components: self-kindness, common humanity, mindfulness, self-judgment, isolation, and over-identification [21]. According to Neff [21], self-kindness represents being supportive and understanding rather than critical toward oneself (i.e., self-judgment). Common humanity refers to recognizing common human experiences and suffering, rather than feeling isolated (i.e., isolation). Mindfulness refers to the awareness and acceptance of one’s internal experiences, rather than being overwhelmed by negative thoughts and emotions (i.e., over-identification). Neff [20] states that the interaction or balance between the positive and negative facets of self-compassion is important, emphasizing that not only the presence of self-compassion but also its absence needs to be viewed in an integrated manner. Therefore, in the present study, we focus not only on the overall effects of self-compassion, but also on the individual effects of its negative (i.e., self-judgment, isolation, and over-identification) and positive components (i.e., self-kindness, common humanity, and mindfulness), as well as their potential synergistic effects [22].

Psychological terms similar to self-compassion include self-esteem, self-affirmation, and self-acceptance. Although self-esteem and self-affirmation involve positive self-evaluation, self-compassion relates to fostering kindness towards oneself even if self-evaluation is negative [23]. Additionally, although both self-acceptance and self-compassion have similar psychological effects, they differ in that self-compassion emphasizes active soothing rather than acceptance of distress [24]. It is assumed that self-compassion is effective in reducing body image concerns by encouraging acceptance of body dissatisfaction while simultaneously promoting proactive efforts to alleviate the associated distress.

Self-compassion micro-interventions, which are ultra-brief, simple exercises that often involve a writing task, have been used with healthy women to prevent eating disorders [18]. However, the effectiveness of these interventions and the best approaches are poorly understood because of the limited number of relevant studies available. A study involving young Japanese women examined the effects of self-compassion interventions on body image concern [18]. During a 30-minute intervention, participants were asked to engage in one of three activities: thinking about words to reduce negative feelings and thoughts about their bodies (i.e., self-compassion micro-intervention), discussing their dissatisfaction with their body, or attending a lecture on cognitive behavior therapy. The results indicated that the self-compassion micro-intervention group showed the most significant improvement in body dissatisfaction. Furthermore, when this intervention was extended to four weeks, participants demonstrated improvements in well-being, appearance schemas, and body image concerns [18]. Another study showed that a 3-minute writing task on self-compassion prevented increased body dissatisfaction due to social media use [25]. These findings suggest that even brief self-compassion exercises may help reduce body dissatisfaction in healthy women. However, it remains unclear which specific components of self-compassion are most effective.

This study aimed to determine the association of each component of self-compassion with body image concerns, eating pathologies, and well-being. Two studies were conducted to clarify these issues. Study 1 used a cross-sectional structural equation modeling (SEM) using survey methodology to identify the most appropriate framework for understanding how self-compassion affects body image and well-being. Study 2 employed a parallel-group randomized controlled trial to examine the effects of the self-compassion micro-intervention on body dissatisfaction, self-esteem, and emotions. More effective interventions could be developed if this study identifies the specific elements of self-compassion that should be enhanced or reduced to address body image concerns. Table 1 presents the definitions of the key terms examined in this study.

**Table 1:** Definitions of the key terms used in this study.

Term		Definition
1	One-factor SC	Represents the overall construct of self-compassion, calculated by reverse-scoring the negative components and summing them with the positive components.
2	Two-factor SC Negative	An indicator of low self-compassion (self-coldness), represented by the total score of self-judgment, isolation, and over-identification.
3	Two-factor SC Positive	An indicator of high self-compassion (self-warmth), represented by the total score of self-kindness, common humanity, and mindfulness.
4	Self-kindness (Six factors)	A positive component of self-compassion, reflecting a kind and gentle attitude toward oneself.
5	Self-judgment (Six factors)	A negative component of self-compassion, reflecting a critical attitude toward oneself.
6	Common humanity (Six factors)	A positive component of self-compassion, reflecting recognition of connection with others in times of suffering.
7	Isolation (Six factors)	A negative component of self-compassion, reflecting a tendency to feel alone and disconnected in times of suffering.
8	Mindfulness (Six factors)	A positive component of self-compassion, reflecting balanced awareness and acceptance of one’s suffering.
9	Over-identification (Six factors)	A negative component of self-compassion, reflecting a tendency to become overly absorbed in one’s suffering.
10	Appearance schemas	Beliefs about the importance of physical attractiveness, which can contribute to maladaptive body image.
11	Body image concern	A maladaptive body image, characterized by negative self-evaluation of appearance, avoidance, and safety behaviors.
12	Abnormal eating	Tendencies associated with eating disorders, including dietary restriction and episodes of overeating.
13	Eudaimonic well-being	The process of achieving well-being, encompassing positive relations with others, autonomy, and personal growth.
14	Hedonic well-being	The outcomes of well-being, including life satisfaction and subjective happiness.

## Study 1

Study 1 investigated the links between self-compassion, well-being, body image concern, and abnormal eating, and developed a path model describing these relationships.

### Method

#### Participants and procedure.

We conducted an online survey after receiving IRB approval (number: 2020-014) from the first author’s affiliated institution. The platform used was Fastask, an online tool that allows researchers to create online survey forms and distribute the questionnaires by setting the target population according to age, gender, place of residence, and other factors. It is a platform many companies and research institutions use for online surveys in Japan. Respondents are rewarded for cooperating with the survey with online points that can be used for online shopping. Informed consent was obtained from all participants included in the study by asking them to click on the check item online. Table 2 describes the participants’ characteristics. In this study, we referred to the guidelines regarding the minimum sample size for structural equation modeling (SEM) [26] and aimed to obtain valid data from more than 500 participants. As a result, Study 1 utilized 604 female community members in Japan from September 16 to December 14, 2020; 577 women completed the survey (response rate: 96%, mean age = 25.1 years, SD = 5.1). Study 1 data were registered with the Open Science Framework (OSF; https://osf.io/yqsjn/?view_only=a52104d4619e48598dccbc56df7b626b).

**Table 2:** Sociodemographic characteristics of the participants.

	Study 1		Study 2					
Characteristics			DN		DNIP		Non-intervention	
	*n*	%	*n*	%	*n*	%	*n*	%
Age								
18─19	140	24.3	10	20.8	15	28.3	20	35.1
20─24	165	28.6	27	56.3	22	41.5	20	35.1
25─29	134	23.2	11	22.9	16	30.2	17	29.8
30─34	138	23.9	0	0.0	0	0.0	0	0.0
BMI								
Underweight	131	22.7	10	20.8	14	26.4	11	19.3
Normal weight	383	66.4	34	70.8	34	64.2	41	71.9
Overweight	37	6.4	4	8.3	5	9.4	5	8.8
Unknown	26	4.5	0	0.0	0	0.0	0	0.0
Marital status								
Single	420	72.8	39	81.3	43	81.1	47	82.5
Married	157	27.2	9	18.8	10	18.9	10	17.5
Children^a^	99	17.2	6	12.5	7	13.2	5	8.8
Employment								
Full-time	229	39.7	25	52.1	13	24.5	12	21.1
Part-time	72	12.5	3	6.3	4	7.5	10	17.5
Housewives	61	10.6	3	6.3	5	9.4	3	5.3
Students	179	31.0	16	33.3	29	54.7	31	54.4
Other	36	6.2	1	2.1	2	3.8	1	1.8
Area of residence								
Urban	187	32.4	7	14.6	18	34.0	11	19.3
Suburban	129	22.4	13	27.1	13	24.5	9	15.8
Rural	261	45.2	28	58.3	22	41.5	37	64.9

DN = decreased negative facet of self-compassion group and DNIP = DN and increased positive facet of self-compassion group.

BMI = body mass index. Underweight (BMI > 18.5 kg/m^2^), normal weight (BMI = 18.5–24.9 kg/m^2^), and overweight (BMI ≥ 25 kg/m^2^) were defined according to the guidelines of the Japan Society for the Study of Obesity [27].

^a^ Reflects the number and percentage of participants answering “yes” to this question.

#### Measures.

We used the following scales, which were demonstrated to have substantial reliability and validity by the developers of each scale.

The Japanese version of the Self-Compassion Scale (J-SCS) is a 26-item scale. The original version (SCS) was developed by Neff [21], which consists of six subscales: self-kindness, common humanity, mindfulness, self-judgment, isolation, and over-identification. It has been suggested that the SCS may be a one-factor model using total scores as well as a six-factor model or a two-factor model consisting of negative and positive factors [28,29]. Example items of the positive component (i.e., self-kindness, common humanity, mindfulness) include “When I experience pain, I am kind to myself,” and “I try to think about my failings as just one part of human being.” Those of the negative component include “When I am having real difficulties, I tend to be hard on myself” and “When I fail at something important to me, I am overwhelmed by feelings of worthlessness.” The items are rated on a five-point Likert scale (1 = “almost never” to 5 = “almost always”). The scores on the negative component were reversed to calculate the total J-SCS scores. Higher levels of positive facets and lower levels of negative facets were associated with low anxiety and depression, high self-esteem and subjective well-being [29]. Both components had negligible correlations with narcissistic personality [29]. The six subscale scores of the J-SCS show substantial internal consistency (.71–.84; [29,30] and 8-week test-retest reliability (.61–.83; [29]). In the present study, Cronbach’s alpha coefficients were .85 for the total score, .87 for the positive and .90 for the negative component. Subscale reliabilities were as follows: Self-Kindness, α = .81; Self-Judgment, α = .81; Common Humanity, α = .69; Isolation, α = .75; Mindfulness, α = .72; and Over-Identification, α = .79. The reliability coefficient for Common Humanity showed a slight tendency to be lower; however, given that it consists of only four items, this result is considered within an acceptable range.

The Brief Psychological Well-Being Scale (BPWBS) is a 24-item scale developed in Japan [31]. It assesses eudaimonic well-being using the six-factor model proposed by Ryff and Singer [32]. The subscales include Autonomy, Self-Acceptance, Positive Relations with Others, Environmental Mastery, Purpose in Life, and Personal Growth. Respondents rated the items (e.g., “In general, I feel positive about myself”) on a six-point Likert scale (1 = strongly disagree, 6 = strongly agree). The composite BPWBS scores were used to assess eudaimonic well-being. The reliability and validity of the BPWBS were previously examined in a Japanese sample of young women. The BPWBS has high internal consistency (.91) and four-week test-retest reliability (.89) [31]. The Cronbach’s alpha in this study was .87. BPWBS scores are inversely correlated with negative emotions and depression and positively associated with positive emotions and hedonic well-being [31]. The score ranged from 24 to 144. Higher scores indicate higher levels of eudaimonic well-being.

The Japanese version of the Subjective Happiness Scale (J-SHS) is a four-item scale that evaluates hedonic well-being [33]. The original SHS was developed by Lyubomirsky and Lepper [34]. An example item is “Some individuals are generally very happy. They enjoy life regardless of what is happening, and get the most out of everything. To what extent does this characterization describe you?” Respondents rated the items on a seven-point scale (1 = “not at all,” 7 = “a great deal”). The reliability and validity of the J-SHS were tested on a sample of young women. The JSHS has high internal consistency (.82) and five-week test-retest reliability of .88 [33]. Although Cronbach’s alpha in this study was .66, the result was considered acceptable given that the scale comprises only four items. J-SHS scores are significantly associated with better health [33]. The score ranges from 4 to 28. Higher scores indicate greater hedonic well-being.

The eight-item self-evaluative salience (SES) subscale of the Japanese version of the Appearance Schemas Inventory-Revised (JASI-R) was used to assess negative body image schemas [35]. The original version was developed by Cash, Melnyk, and Hrabosky [36]. Example items include “When I meet people for the first time, I wonder what they think about how I look,” and “If I dislike how I look on a given day, it’s hard to feel happy about other things.” All items were rated on a five-point scale from 1 (“strongly disagree”) to 5 (“strongly agree”). The original SES subscale is a 12-item inventory [35]. SES is an eight-item subscale in the Japanese version while the original version consists of 12 items. The following four items were discarded because of low loadings on the SES factor: “I seldom compare my appearance to that of other people I see,” “If somebody had a negative reaction to what I look like, it wouldn’t bother me,” “My physical appearance has had little influence on my life, ” and “By controlling my appearance, I can control many of the social and emotional events in my life.” However, the original version used principal components analysis with varimax rotation while the Japanese version used factor analysis with a maximum-likelihood method and Promax rotation. Different analytic methods may explain the different results. The SES subscale has good reliability (a = .82; four-week test-retest reliability = .75) [35]. Cronbach’s alpha in this study was .84. The SES is significantly convergent with other measures of body image and psychosocial functioning [35]. The score ranges from 13 to 40. High scores indicate a high investment in one’s appearance for self-evaluation.

We used the Japanese version of the Body Image Concern Inventory (J-BICI), which assesses body image disturbances other than body image schemas, such as body dissatisfaction, emotional distress, and behavioral problems [37]. The J-BICI is a 19-item scale consistent with the original version developed by Littleton, Axsom, and Pury [5]. However, a study that examined the factor structure of the J-BICI, adjusting for sex differences, found that a three-factor model of the J-BICI is the most interpretable: Negative Evaluation of one’s own appearance, Safety Behaviors regarding one’s own appearance flaws, and Avoidant Behaviors concerning one’s own appearance flaws. The original version comprised two factors: Dysmorphic Appearance Concern and Interference due to Appearance Concerns. As Littleton et al. [5] recommend, the total score of all subscales was used in this study. Example items include “I am dissatisfied with some aspect of my appearance,” and “I try to camouflage certain flaws in my appearance.” The items are rated on a five-point Likert scale from 1 (never) to 5 (always). The total scores on the J-BICI have good internal consistency (.91) and three-week test-retest reliability (.88) [37]. Cronbach’s alpha in this study was .93. The total scores on the J-BICI are significantly convergent with depression, obsessive-compulsive tendencies, eating pathology, and other measures of body image [37]. Scores range from 19 to 105. High scores reflect high body image concerns.

The Abnormal Eating Behavior Scale-New version (AEBS-NV) is a 14-item scale developed with female university students in Japan [38]. The AEBS-NV consists of three factors: Inappropriate Dieting Behavior, Apprehension Concerning Food Intake, and Binge Eating. The composite AEBS-NV scores were used in this study to assess abnormal eating tendencies. Example items include “I want to stop being obsessed with food,” and “I tolerate any dieting if I can avoid being fat.” Participants rate each item on a six-point scale from 1 (“strongly disagree”) to 5 (“strongly agree”). This scale has good reliability (.84–.89) [38]. Cronbach’s alpha in this study was .94. Women who score higher on the AEBS-NV exhibit significantly greater eating pathology than women who score lower [38]. Scores range from 7 to 35, with higher scores indicating more abnormal eating.

The self-reported questionnaire asked the respondents’ age, height, weight, residence area, employment situation, marital status, and presence or absence of children. Self-reported height and weight were used to determine body mass index (BMI; i.e., weight in kilograms divided by squared height in meters). Self-reported height and weight has shown to correlate with objectively measured values in adults [39,40].

#### Analytic strategy.

Amos 29 was used in this study. The following cut-off values were used to examine whether the result of SEM was a good fit: GFI of .95 or more [41], CFI and NNFI of .95 or more, RMSEA of .06 or less, SRMR of .08 or less [42]. Hypothetical models for SEM were developed: Since the review study suggested that self-compassion affects well-being [16], we drew a path from one-, two-, and six-factor self-compassion to well-being. Well-being was considered a latent variable consisting of eudaimonic and hedonic well-being. It has also been shown that self-compassion impacts negative body image variables [17]. Therefore, we drew paths from one-, two-, and six-factor self-compassion to appearance schemas, body image concern, and abnormal eating. In Japan, self-compassion’s negative and positive components are independent [29,30]. Therefore, we did not assume an association between them.

Paths were drawn based on the cognitive-behavioral model of body image for associations between variables related to body image [10]. Notably, we drew paths from the appearance schema to body image concern and abnormal eating [6,14,15]. Furthermore, because well-being is a construct that encompasses both hedonic and eudaimonic aspects [32], it was treated as a latent variable in this study to capture its multifaceted nature while controlling for measurement error. Well-being can serve as an outcome and a protective factor against negative body image. Several studies have emphasized improving well-being when treating body image concerns. For instance, research examining the relationships between well-being, eating disorders, and body dissatisfaction in adolescents found that the domains of life satisfaction and engagement had a positive impact on psychological health [43]. Increasing well-being has been identified as an effective complement to cognitive-behavioral interventions for decreasing body image concerns [44]. It is also crucial for preventing relapses in eating disorders [45–47]. Thus, we added paths from well-being to body image concerns and abnormal eating habits.

### Results

#### Relationships among self-compassion, well-being, body image concern, and abnormal eating.

Tables 3 and 4 present the Pearson’s correlation coefficients. One-factor self-compassion was significantly associated with negative body image and well-being. When the self-compassion scores were divided into two factors, only the negative component was significantly correlated with negative body image. Both components of self-compassion were significantly correlated with well-being.

**Table 3:** Summary of correlations regarding one- and two-factor self-compassion.

	Variable	1	2	3	4	5	6	7	8
1	One-factor SC	—							
2	Two-factor SC Negative	**–.76** ^***^	—						
3	Two-factor SC Positive	**.65** ^***^	.01	—					
4	Eudaimonic well-being	**.58** ^***^	**–.39** ^***^	**.45** ^***^	—				
5	Hedonic well-being	**.45** ^***^	**–.33** ^***^	**.31** ^***^	**.58** ^***^	—			
6	Appearance schemas	**–.37** ^***^	**.57** ^***^	**.10** ^*^	**–.23** ^***^	**–.21** ^***^	—		
7	Body image concern	**–.32** ^***^	**.45** ^***^	.04	**–.30** ^***^	**–.33** ^***^	**.59** ^***^	—	
8	Abnormal eating	**–.14** ^***^	**.24** ^***^	.06	**–.21** ^***^	**–.26** ^***^	**.34** ^***^	**.66** ^***^	—
	*M*	74.7	42.15	38.85	16.65	26.11	55.73	39.45	84.53
	*SD*	13.07	9.96	8.45	4.22	6.1	15.14	15.5	14.84

N = 577; SC = self-compassion; Bold indicates significant paths, and non-significant paths are shown in gray.

***p < .001, *p < .05.

**Table 4:** Summary of correlations regarding six-factor self-compassion.

	Variable	1	2	3	4	5	6
Six-factor SC							
1	Self-kindness	—					
2	Self-judgment	**–.15** ^***^	—				
3	Common humanity	**.57** ^***^	.03	—			
4	Isolation	–.01	**.65** ^***^	**.10** ^*^	—		
5	Mindfulness	**.60** ^***^	.04	.61^***^	.05	—	
6	Over-identification	–.05	**.73** ^***^	.01	**.67** ^***^	.05	—
Eudaimonic well-being		**.41** ^***^	**–.36** ^***^	**.32** ^***^	**–.37** ^***^	**.41** ^***^	**–.30** ^***^
Hedonic well-being		**.29** ^***^	**–.28** ^***^	**.21** ^***^	**–.34** ^***^	**.27** ^***^	**–.25** ^***^
Appearance schemas		.01	**.50** ^***^	**.14** ^**^	**.50** ^***^	**.13** ^**^	**.52** ^***^
Body image concern		–.04	**.42** ^***^	**.09** ^*^	**.39** ^***^	.07	**.40** ^***^
Abnormal eating		.02	**.20** ^***^	**.13** ^**^	**.29** ^***^	.02	**.17** ^**^
	*M*	15.37	16.61	11.52	12.1	11.96	13.44
	*SD*	3.87	4.19	3.08	3.46	2.95	3.56

N = 577; Bold indicates significant paths, and non-significant paths are shown in gray.

***p < .001, **p < .01, *p < .05.

#### Verification of the hypothetical models.

**Fig 1:**
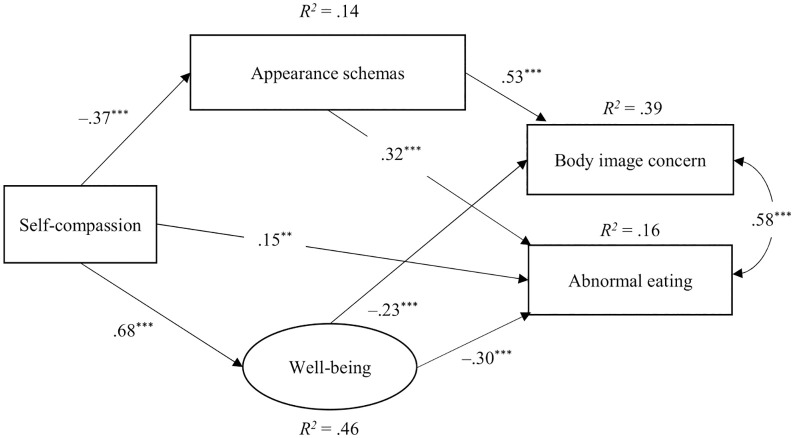
A path model of one-factor self-compassion. ^***^p < .001, ^**^p < .01; Nonsignificant paths omitted.

Tables 4–6 demonstrate the results of SEM. In Model 1, in which one-factor self-compassion was an observed variable, the path from self-compassion to body image concern was not significant (β = .08, p = .165). Consequently, we deleted this path in Model 2 and conducted SEM. The resultant goodness-of-fit indices for Model 2 were substantial (Fig 1). In Model 3, in which self-compassion was a latent variable, the paths from well-being to body image concern (β = –.09, p = .185) and from well-being to abnormal eating (β = –.02, p = .769) were not significant. The goodness-of-fit indices remained low after removing these nonsignificant paths in Model 4. These results support the validity of Model 2 (Fig 1). The model in Fig 1 demonstrated that self-compassion significantly predicted lower appearance schema and higher well-being. Additionally, through appearance schemas and well-being, self-compassion indirectly influenced body image concerns and abnormal eating.

**Table 5:** Structural equation modeling results of each self-compassion model.

Model		χ^2^	*df*	CFI	TLI	RMSEA [90%CI]	SRMR	AIC	BIC
One-factor SC									
1	SC as observed variable	12.42^*^	5	.994	.981	.051 [.015, .087]	.028	44.42	114.15
2	SC as observed variable (without NSP)	10.52^*^	4	.995	.979	.053 [.014, .094]	.025	44.52	118.60
3	SC as latent variable	357.88^***^	32	.886	.804	.133 [.121, .146]	.147	425.88	574.05
4	SC as latent variable (without NSP)	359.42^***^	34	.886	.816	.129 [.117, .141]	.147	423.42	562.87
Two-factor SC									
5	SC as observed variable	14.74^*^	6	.994	.977	.050 [.018, .083]	.028	58.74	154.62
6	SC as observed variable (without NSP)	21.19^**^	8	.990	.974	.053 [.026, .082]	.029	61.19	148.35
7	SC as latent variable	293.11^***^	35	.910	.858	.113 [.101, .125]	.110	355.11	490.20
8	SC as latent variable (without NSP)	301.05^***^	37	.908	.863	.111 [.100, .123]	.110	359.05	485.43
Six-factor SC									
9	SC as observed variable	93.34^***^	24	.976	.944	.071 [.056, .086]	.045	177.34	360.37
10	SC as observed variable (without NSP)	105.81^***^	31	.974	.954	.065 [.051, .078]	.044	175.81	328.33

SC = self-compassion and NSP = non-significant path(s).

***p < .001, **p < .01, *p < .05.

**Table 6:** Key significant pathways in the final models of self-compassion.

Model	χ^2^	*df*	CFI	TLI	RMSEA [90%CI]	SRMR	Kay significant paths (β)
One-factor SC							
SC as observed variable (without NSP)	10.52^*^	4	.995	.979	.053 [.014, .094]	.025	SC → Appearance schemas (–.37^***^), Abnormal eating (.15^**^), and WB (.68^***^)
Two-factor SC							
SC as observed variable (without NSP)	21.19^**^	8	.990	.974	.053 [.026, .082]	.029	Negative facet of SC → Appearance schemas (.57^***^) and WB (–.47^***^); Positive facet of SC → Appearance schemas (.10^**^), body image concern (.16^***^), abnormal eating (.20^***^), and WB (.51^***^)
Six-factor SC							
SC as observed variable (without NSP)	105.81^***^	31	.974	.954	.065 [.051, .078]	.044	Self-kindness → WB (.24^***^); Self-judgment → Appearance schemas (.19^***^) and WB (–.18^***^); Common humanity → Appearance schemas (.12^***^) and abnormal eating (.10^**^); Isolation → Appearance schemas (.20^***^), abnormal eating (.14^**^), and WB (–.35^***^); Mindfulness → WB (.33^***^); Over-identification →Appearance schemas (.25^***^) and abnormal eating (–.19^***^)

SC = self-compassion, WB = well-being, and NSP = non-significant path(s).

***p < .001, **p < .01, *p < .05.

**Fig 2:**
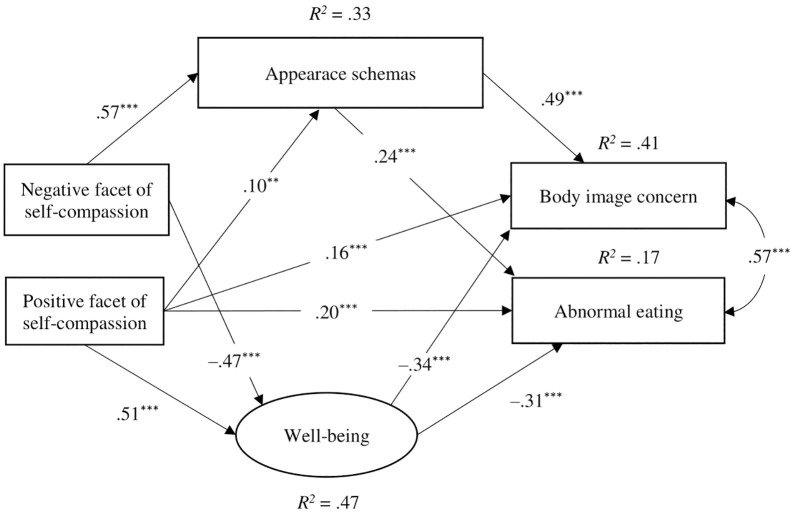
A path model of two-factor self-compassion. ^***^p < .001, ^**^p < .01; Nonsignificant paths omitted.

In Model 5, with two-factor self-compassion as the observed variable, the paths from the negative facet to body image concern (β = –.04, p = .399) and abnormal eating (β = –.08, p = .173) were not significant. After removing these paths, SEM was performed for Model 6 and the goodness-of-fit indices were substantial (Fig 2). In Model 7, the paths from the negative facet to body image concerns (β = .06, p = .266) and abnormal eating (β = –.09, p = .138) were nonsignificant. Even after deleting these paths, the goodness-of-fit indices were low in Model 8. These findings supported the validity of Model 6 (Fig 2). Fig 2 indicates that the negative facet significantly predicted higher appearance schemas and well-being and indirectly predicted body image concerns and abnormal eating behaviors through these variables. The positive facet significantly predicted higher well-being and higher appearance schemas, body image concerns, and abnormal eating behaviors. Our results showed that the negative facet has adaptive and maladaptive effects on body image and eating behaviors.

In Model 9, with six-factor self-compassion, several paths were not significant: from common humanity to well-being (β = .08, p = .086), self-kindness to appearance schemas (β = –.06, p = .157), mindfulness to appearance schemas (β = .07, p = .157), over-identification to well-being (β = .01, p = .828), self-kindness to abnormal eating (β = .04, p = .317), self-judgment to abnormal eating (β = –.08, p = .093), and mindfulness to abnormal eating (β = –.07, p = .100).

**Fig 3:**
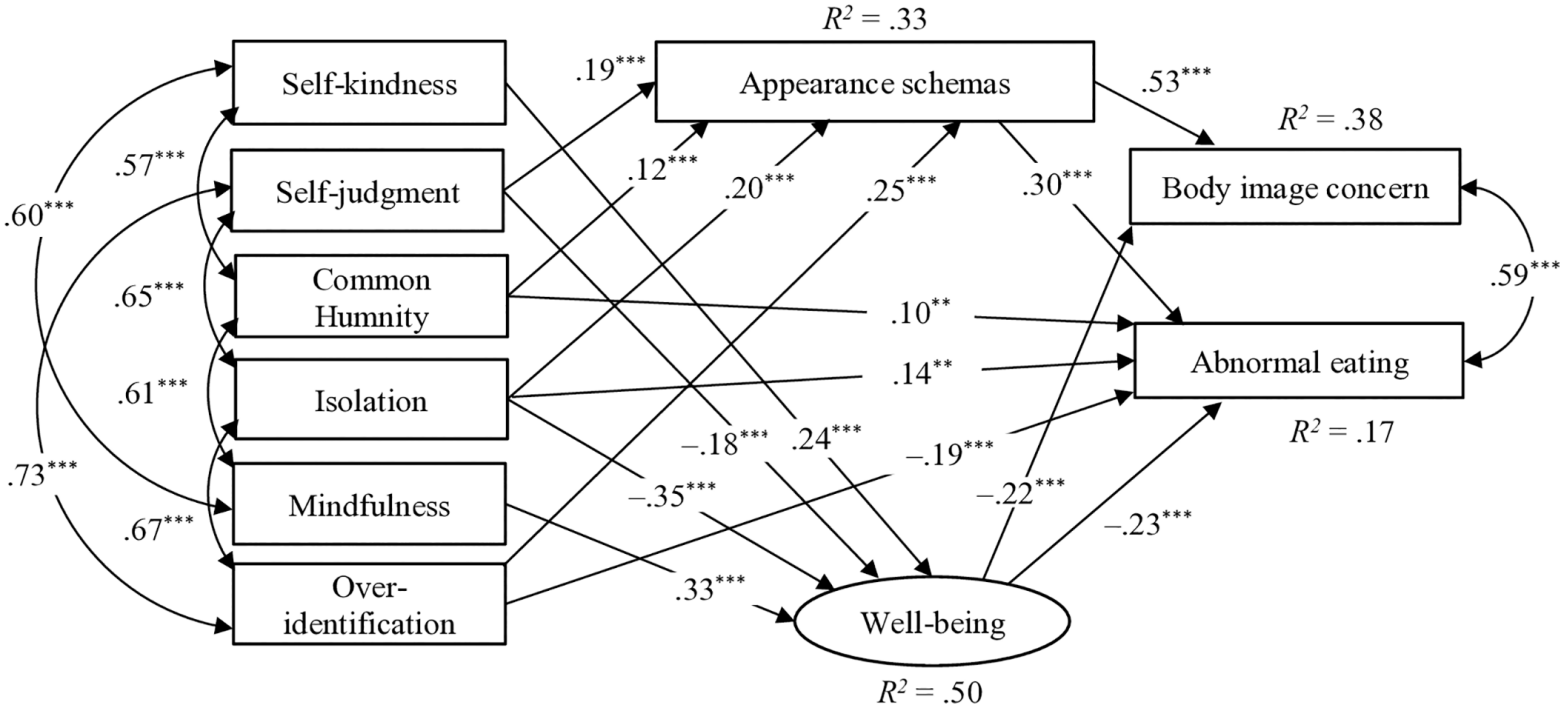
A path model of six-factor self-compassion. ^***^p < .001, ^**^p < .01; Nonsignificant paths omitted.

We deleted these paths; however, the goodness-of-fit index RMSEA did not meet the criteria for an acceptable fit. Thus, the validity of the six-factor self-compassion model (Fig 3) was not supported.

### Discussion

This study explored the relationship between self-compassion, well-being, appearance schemas, body image concerns, and abnormal eating. Further, it sought to develop a model that describes how self-compassion predicts well-being and body image-related variables.

Analysis of the relationships between the self-compassion constructs revealed no significant correlations between the negative and positive components of self-compassion. Similarly, no significant associations were observed between self-kindness and self-judgment, common humanity and isolation, or mindfulness and overidentification, which are considered counter-concepts [21]. These findings are consistent with previous Japanese samples studies [29,30]. Research comparing the relationships between the positive and negative elements of self-compassion across countries has also identified variations irrespective of Western or Eastern cultural distinctions [28]. Ishimura [30] suggested that the negative and positive aspects of self-compassion coexist among Japanese women. This coexistence may reflect the tendency for Japanese women to hold both positive and critical views of their bodies, potentially contributing to internal conflict.

This study also found that higher levels of the positive component of self-compassion and lower levels of the negative component were associated with greater well-being. This finding aligns with a meta-analysis that demonstrated a link between self-compassion and well-being [16]. Additionally, the study revealed that individuals with higher levels of the negative aspect of self-compassion tended to place greater emphasis on external attractiveness, which in turn heightened their body image concerns and tendencies toward eating disorders. These results are consistent with previous studies [17,18,25].

In this study, a significant positive correlation was found between mindfulness and appearance schemas. The practice of mindfulness may enhance awareness of how important physical appearance is to the self, making this result understandable. Even if individuals place a high value on appearance, having greater mindfulness may help mitigate its negative effects [18]. On the other hand, the significant positive correlation between common humanity and body image-related variables was an unexpected finding that contradicted previous research [17,18,25]. In the context of Japanese collectivistic culture, the idea expressed by common humanity may not provide comfort, but rather evoke a sense of pressure, such as “if others are enduring hardship, I must push myself even harder.” Additionally, no significant correlation was found between self-kindness and body image variables. This may be due to the ambivalence and guilt that Japanese women may experience when they feel they do not meet socially expected appearance standards and yet try to treat themselves with kindness. These findings suggest that both the assessment and application of self-compassion among Japanese women require cultural sensitivity and careful consideration.

The comparison of self-compassion models showed that both the one-factor model and two-factor model were valid. Scores from the one-factor model, as well as the negative components in the two-factor model, significantly predicted well-being and appearance schemas, which in turn predicted body image concerns and disordered eating behaviors. These findings support the hypotheses of this study and align with the cognitive-behavioral model of body image [10]. In contrast, the positive components in the two-factor model significantly predicted undesirable outcomes related to body image. This result contradicts earlier studies [17,18,25], highlighting a complex finding.

Based on these results, interventions aimed at increasing self-kindness, common humanity, and mindfulness among Japanese women should be designed with care, ensuring that these positive attitudes do not inadvertently intensify self-criticism. As Markus and Kitayama [48] have noted, Western cultures tend to promote an independent self, whereas East Asian cultures emphasize an interdependent self—one that values connection with others, social harmony, and fulfilling social roles. Accordingly, many Japanese women may develop negative body image not only due to internalized ideals, but because they feel they are not meeting external appearance standards expected by society or others. In such cultural contexts, a message like “many others are struggling too,” rooted in common humanity, may not bring comfort, but instead heighten social comparison and conformity pressure. Therefore, when designing body image interventions for Japanese young women, it may be more effective to focus on reducing the negative components of self-compassion, while also recognizing that the positive and negative components interact synergistically to improve mental health outcomes [49].

## Study 2

Although Study 1 examined the impact of self-compassion on body image and well-being using a cross-sectional design, it is challenging to identify causal relationships in cross-sectional studies. Thus, Study 2 employed a parallel-group randomized controlled trial to examine the synergistic effects of combining a decrease in the negative component and an increase in the positive component of self-compassion, through a self-compassion micro-intervention, on body dissatisfaction, self-esteem, and emotional states. The present study examined the following hypotheses: While decreasing the negative component alone is expected to reduce body dissatisfaction and negative emotions and increase self-esteem and positive emotions [18], combining a reduction in the negative component with an increase in the positive component is expected to lead to even greater improvements, as suggested by the model of Study 1 (Figs 1 and 2).

### Method

#### Participants and procedure.

We conducted an online survey after receiving IRB approval (number: 2021-128) from the first author’s affiliated institution. The platform used was Fastask, as in Study 1. Informed consent was obtained from all participants included in the study by asking them to click on the check item online. Table 1 describes the participants’ characteristics.

Based on an assumed effect size of 0.2, a G*Power analysis indicated that 141 participants were required. However, since this was an online survey with several open-ended tasks, we expected the valid response rate to drop to about 40%. To ensure we would still have at least 141 valid responses after excluding invalid ones, we recruited more participants than the required sample size. The survey was administered to 348 women from August 25 to September 15, 2021. The participants were randomly assigned to one of the following three groups: decreased negative facet of self-compassion group (DN group), DN combined with increased positive facet of self-compassion group (DNIP group), and non-intervention group. Participants first completed an image task [50,51] to increase state-negative body dissatisfaction. Upon completion, we directed them to complete the pre-test, which included the measurement of state body dissatisfaction, self-esteem, positive emotion, and negative emotion. Subsequently, the DN and DNIP groups completed self-compassion tasks while the non-intervention group completed a control task. After the completion, participants in all groups answered the post-test, measuring state body dissatisfaction, self-esteem, positive emotion, negative emotion, and demographic items. Participation in the study lasted approximately 20 minutes.

**Fig 4:**
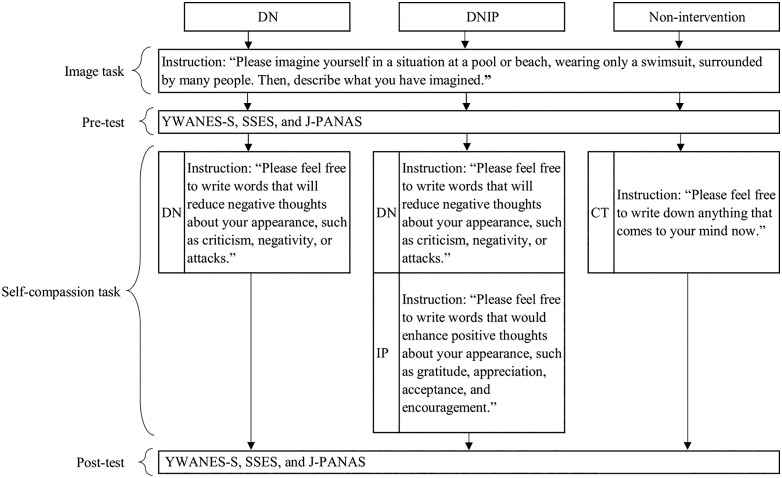
Overview of the experimental procedure and task instructions in Study 2. YWANES-S = Young Women’s Appearance-Related Negative Emotions Scale -State Version, SSES = State Self-Esteem Scale, J-PANAS = Japanese version of the Positive and Negative Affect Schedule, CT = control task.

The final sample sizes that successfully completed the experimental tasks were 48 participants in the DN group (mean age 22.6 years, SD = 3.1), 53 in the DNIP group (mean age = 22.8 years, SD = 3.6), and 57 in the non-intervention group (mean age = 22.4 years, SD = 3.5). The final number of valid responses decreased from 348 to 158. Study 2 data was registered to OSF: https://osf.io/t39ue/?view_only=c89a2f185fe4412e8ce75c47e4bde1a9. Fig 4 illustrates the protocol for Study 2, which is also available on protocols.io (DOI: https://doi.org/10.17504/protocols.io.8epv5214jv1b/v1).

#### Measures.

State body dissatisfaction was assessed using the “Young Women’s Appearance-Related Negative Emotions Scale -State Version” [50]. This scale comprises seven items reflecting a single factor structure. Participants respond to the items describing negative feelings toward one’s physical appearance, such as “sad” and “disappointed” on a five-point scale, where 1 indicates “not applicable at all” and 5 indicates “very applicable.” The scale demonstrated high internal consistency with a Cronbach’s alpha coefficient of .94 [50]. Cronbach’s alpha of this study’s data was .92 for the pre-test and .94 for the post-test. It has shown significant correlations with trait body dissatisfaction, state self-esteem, depression, anxiety, and fatigue (r = .59, r = −.66, r = .49, r = .43, r = .42, all p < .001 [50]). Furthermore, individuals engaging in a task designed to increase body dissatisfaction temporarily exhibited significantly higher scores compared to those who did not engage in the task (p < .001), confirming the scale’s reliability and validity. The total scores ranged from seven to 35, were used in this study, with higher scores indicating greater state somatic dissatisfaction.

State self-esteem was measured using the State Self-Esteem Scale [52], a modified state version of the Japanese Rosenberg’s Self-Esteem Scale [53]. The scale includes nine items reflecting a single-factor structure. Participants responded to items such as “I feel that I am as valuable as other people now” on a five-point scale ranging from 1 (not applicable) to 5 (applicable). The scale exhibited good internal consistency with a Cronbach’s alpha coefficient of .83. Cronbach’s alpha of this study’s data was .80 for the pre-test and .74 for the post-test. The reliability and validity of the scale were supported by its responsiveness to feedback, with scores increasing following positive feedback and decreasing with negative feedback [52]. In this study, the total score was used, ranging from nine to 45, where higher scores indicate higher state self-esteem.

Emotional states were assessed using the Japanese version of the Positive and Negative Affect Schedule (J-PANAS) [54], adapted from the original scale developed by Watson, Wiese, Vaidya, and Tellegen [55]. This scale features a two-factor structure with ten items for each factor. Participants rated the extent to which words such as “motivated” (positive affect) and “embarrassed” (negative affect) described their current mood on a 5-point scale ranging from 1 (not at all applicable) to 6 (very applicable). The J-PANAS demonstrated strong internal consistency with Cronbach’s alpha coefficients ranging from .85 to .86 for positive affect and .88 to .89 for negative affect [54]. Cronbach’s alpha for the positive and negative affect of this study’s data was .93 and 91 for the pre-test and .94 and .92 for the post-test. Positive affect was negatively correlated with low positive affect (r = –.47; p < .01), and negative affect was positively correlated with depression (r = .60; p < .01), confirming the scale’s reliability and validity. The total scores for each factor, ranging from 10 to 50 points, were used in this study, with higher scores indicating a higher level of the relevant emotional state.

The self-reported questionnaire asked for the respondents’ age, height, weight, residence area, employment situation, marital status, and presence or absence of children.

Self-reported height and weight were used to determine BMI.

#### Experimental design and conditions.

This randomized controlled trial had self-compassion as the independent variable and body dissatisfaction, self-esteem, and emotions as the dependent variables. Regardless of the group to which they were assigned, the participants were told that the study was a survey about themselves that included questions about their current physical and mental state. The experiment involved two cognitive tasks: an image task and a self-compassion task. These cognitive tasks were also presented as an open-ended survey as part of the questions regarding participants’ physical and mental states.

This study used an image task (Fig 4) shown to increase body dissatisfaction [50,51]. Participants were instructed to imagine a situation at a pool or beach, wearing only a bathing suit, with many people around them. An illustration of a woman wearing a bathing suit on the beach is presented to aid visualization. In addition, they were asked to report what they had imagined. This report served as a manipulation check for the image tasks. Women who reported irrelevant images were considered to have failed to perform the task adequately and were excluded from the analysis. Baseline assessments were conducted after temporarily increasing body dissatisfaction using the image task.

**Fig 5:**
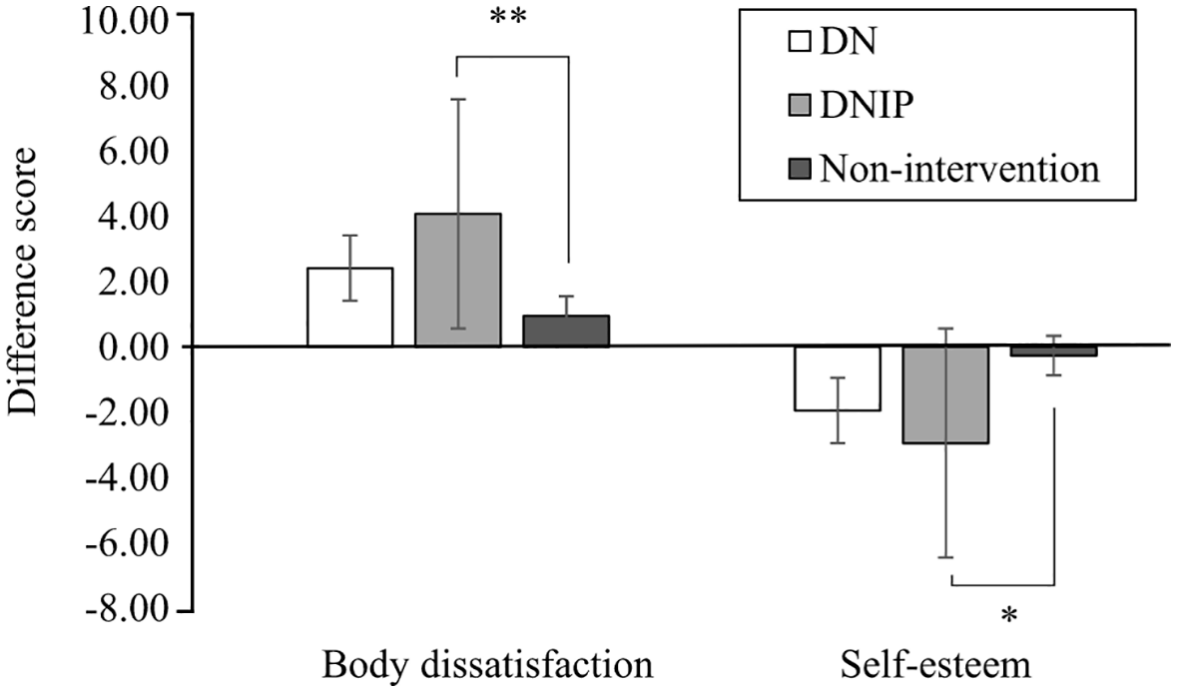
Difference scores for body dissatisfaction and self-esteem in each group. DN = decreased negative facet of self-compassion group and DNIP = DN and increased positive facet of self-compassion group.

The self-compassion task (Fig 4) was intended to increase or decrease self-compassion’s positive and negative components. The written task was created based on compassionate mind training (CMT) [18], effectively reducing negative body image and increasing well-being among young Japanese women. CMT includes a written task that asks participants to think of words that help reduce negative feelings and thoughts about the body. In the present study, this task was used to reduce the negative elements of self-compassion in the DN and DNIP groups. The participants received the prompt: “Please feel free to write words that will reduce negative thoughts about your appearance, such as criticism, negativity, or attacks.” Example reports in the DN and DNIP groups were “You are your own person so do not compare yourself to others,” “Each person is different, so do not think too bad,” and “Blaming myself is not good.” In addition to this task, the DNIP group completed a written task asking them to think of words that would help them increase their positive feelings and thoughts about their bodies. The participants received the prompt: “Please feel free to write words that would enhance positive thoughts about your appearance, such as gratitude, appreciation, acceptance, and encouragement.” Example reports were “it is not your fault,” “others are more worried about their appearance than me,” and “accept things as they are.” In the non-intervention group, participants were given a neutral prompt: “Please feel free to write down anything that comes to your mind now.” Examples of statements from the non-intervention group were “I need to lose my weight to go to the beach or pool,” “I would look more attractive if I lost my weight,” and “I looked at the Instagrams of my friends who went to the pool and the beach and felt envious of their good look.”

The responses to the self-compassion task were used as manipulation checks. Women who responded with a meaningless description (e.g., aaa) or “I don’t know” in the description section of the subsequent DN, DNIP, and non-intervention tasks were also considered to have failed to perform the task adequately and were excluded from the analysis. Given that it is challenging to identify the personal function of a statement from its content, all data from women who provided meaningful descriptions were included in the analysis, regardless of the content.

#### Analytic strategy.

One-way analysis of variance (ANOVA) was performed using SPSS Statistics 29 with the significance level set at 5%. Effect size interpretation followed the guidelines by Statology [56], with η² = .01 indicating a small effect, η² = .06 a medium effect, and η² = .14 or greater considered a large effect. The dependent variables were the change scores obtained by subtracting the post-test value from the pre-test scores of negative body image state, self-esteem state, positive emotions, and negative emotions. Positive difference scores for body dissatisfaction and negative affect suggest a reduction in body dissatisfaction over time, from pre-to post-task. In contrast, negative difference scores for self-esteem and positive affect reflected an increase in both self-esteem and positive affect over the same period.

### Results

A one-way analysis of variance (ANOVA) was conducted to examine the effect of the groups on negative body image state, self-esteem state, positive emotion, and negative emotion. No significant differences were observed in the means of age and BMI between the groups (p = .820, .847, respectively). The result showed a significant main effect of the groups on negative body image state (Fig 5; F(2, 155) = 5.02, p = .008, η^2 ^= .061). Post-hoc comparisons using the Bonferroni method indicated that the DNIP group differed significantly from the non-intervention group (p = .006). Simultaneously, no significant difference was found between the DN and the non-intervention groups (p = .447). The analysis also revealed a significant main effect of the groups on state self-esteem (Fig 5; F(2, 155) = 4.57, p = .012, η^2^ = .056). Post-hoc comparisons using the Bonferroni method indicated that the DNIP group differed significantly from the non-intervention group (p = .010). Simultaneously, no significant difference was found between the DN and the non-intervention groups (p = .213). The results showed no significant main effect of the groups on positive (F(2, 155) = 1.39, p = .253, η^2^ = .018) and negative emotions (F(2, 155) = 0.06, p = .942, η^2^ = .001), while the effect size of positive emotion was small.

### Discussion

This study examined the short-term synergistic effects of combining the reduced negative and the increased positive components of self-compassion on well-being and body image. The intervention, which increased the positive component while reducing the negative component, significantly decreased body dissatisfaction. However, it had no significant impact on positive or negative emotional states and reducing the negative facet alone did not affect body dissatisfaction or self-compassion. These findings did not support our hypothesis.

There are several possible explanations for why the intervention in this study did not lead to significant changes in positive and negative emotions as measured by the J-PANAS. It is possible that the J-PANAS was not sufficiently sensitive to detect the effects of the intervention. While changes were observed in measures assessing self-directed emotions, detecting change may have been more difficult with a general affect measure. Furthermore, considering that Study 1 supported a model in which self-compassion significantly predicted well-being, it is also plausible that the brief nature of the current intervention was insufficient to induce broader emotional changes. Future research may yield more meaningful results by allowing participants to engage more thoroughly with the self-compassion tasks which may produce significant changes in J-PANAS scores.

According to models in Study 1, self-compassion improves body image through enhanced well-being, which corresponded to self-esteem and emotional states in this study. Previous studies [18,25] have shown that self-compassion interventions can reduce body dissatisfaction. However, the individual effects of reducing the negative aspects of self-compassion, as well as the additional effects of simultaneously enhancing its positive aspects, have not been sufficiently investigated. The results of this study are considered to support previous research [48], which suggested that reducing the negative components of self-compassion and enhancing its positive components help regulate individuals’ balancing systems and contribute to the effectiveness of self-compassion interventions. Further research is needed to elucidate the mechanisms underlying these combined effects.

This study utilized an online survey to deliver a brief self-compassion intervention, but a key limitation was the reduced completion rate associated with this method. One possible explanation is that since many participants used smartphones to answer, the psychological burden of text entry may have been high, leading to invalid responses. The final sample, who appropriately completed the open-ended tasks in this study, were likely highly motivated to participate and demonstrated a strong ability to understand and respond to the tasks accurately, even in an online environment where real-time questioning was not possible. These participant characteristics may have positively influenced the results of the study.

It is crucial to develop psychological interventions that are both time-efficient and effective in online settings to enhance accessibility for both facilitators and participants [57]. Future studies should consider incorporating verbal explanations of the intervention, such as video demonstrations, or allowing participants to provide verbal responses via recording functions instead of written input. These adjustments could improve both comprehension and task completion rates.

Additionally, the content of the descriptions was used as a manipulation check for the self-compassion task in this study. However, the lack of a formal fidelity check in this study may have affected the content validity. It will be necessary to conduct independent coding of the descriptions and calculate inter-rater reliability in future research to examine the validity of the manipulation check.

Another limitation of this study is that we could not fully control for the demand effect [58]. In this study, to control for demand effects, participants were randomly assigned to each condition, and the purpose of the study was explained to all groups as a survey regarding physical and mental health. The experimental task was designed as an open-ended survey to determine participants’ physical and mental states. However, it remains possible that participants in the DN and DNIP groups may have inferred that the researchers expected positive changes in body dissatisfaction. In future research, although the non-intervention group will not focus on self-compassion, tasks that encourage more active involvement, such as having participants write about ways to cope with body dissatisfaction, may help better control for demand effects.

## General discussion

The present study aimed to determine how the negative and positive components of self-compassion are related to well-being, appearance schemas, body image concerns and eating pathology.

In the present study, we conducted two studies. Study 1 showed that the one- and two-factor models of self-compassion were valid in explaining well-being and maladaptive body image. Notably, in the two-factor model, contrary to expectations, a higher level of the positive component significantly predicted higher levels of maladaptive appearance-related schemas, body image concern, and abnormal eating behaviors. However, Study 2 showed that a reduction in the negative component combined with an increase in the positive component had an adaptive influence on body image and self-esteem. This study suggests that verbalizing statements that reduce the negative and enhance the positive facets of self-compassion may be helpful in addressing body image concerns and maladaptive eating behaviors.

In conclusion, this study suggests that both the positive and negative elements of self-compassion collectively influence body image, rather than through specific individual components. However, the mechanisms underlying these effects remain unclear. Future research should explore the factors contributing to these synergistic effects and investigate whether the daily exercise of the self-compassion intervention used in this study leads to improvements in well-being, appearance schemas, body image concerns, and disordered eating behaviors.

The findings of the present study have important practical implications. Specifically, they may be incorporated into health education curricula for adolescents and mental health training programs for educators working with youth as a simple and accessible coping strategy to manage body image–related stress. Furthermore, in digital health programs targeting adults, including guidance that encourages individuals to stop criticizing their bodies and instead focus on positive self-statements could contribute to the maintenance and improvement of a healthy body image. These applications highlight the potential for translating research findings into interventions across different age groups and settings, promoting healthy body image and well-being through enhanced self-compassion.
